# The Aseptic Finger as a Tourniquet

**Published:** 1897-06-05

**Authors:** 


### THE ASEPTIC FINGER AS A TOURNIQUET.

The fact that a clean finger can be introduced into the peritoneum and kept there for a considerable period without incurring any risk of intraperitoneal sepsis, is a matter which bears fruit outside the bounds of abdominal surgery. It is not difficult to call to mind many circumstances under which temporary access to parts which can only be got at through the peritoneum may be of great utility. One of these is the transperitoneal ligature of the iliac artery. Now, however, Dr. Charles McBurney advocates the introduction of the finger within the abdomen to control the common iliac artery during amputation of the hip joint. An incision is made through the skin about one and a half inches internal to the anterior spine of the ilium, and is carried down by separating the aponeurotic and muscular fibres as in the operation for removal of the appendix between the attacks. Through this incision an assistant passes his index finger which readily reaches and controls the common iliac artery. Not only does this proceeding give the advantage of complete control of the blood current during the operation, but it leaves the whole of the operative field free from bandage or appliance of any kind, and thus gives full opportunity of maintaining complete asepsis. Another advantage is that it becomes easy after the limb is removed to identify and ligature all the smaller arteries in the stump, as by raising the finger for a moment any vessel which is unligatured can be made to show. Three cases are reported, all of which did well.

